# Sex, gender and work segregation in the cultural industries

**DOI:** 10.1111/1467-954X.12238

**Published:** 2015-05-26

**Authors:** David Hesmondhalgh, Sarah Baker

**Keywords:** work segregation, cultural industries, stereotypes, sexual division of labour

## Abstract

This chapter addresses work ‘segregation’ by sex in the cultural industries. We outline some of the main forms this takes, according to our observations: the high presence of women in marketing and public relations roles; the high numbers of women in production co-ordination and similar roles; the domination of men of more prestigious creative roles; and the domination by men of technical jobs. We then turn to explanation: what gender dynamics drive such patterns of work segregation according to sex? Drawing on interviews, we claim that the following stereotypes or prevailing discourses, concerning the distinctive attributes of women and men, may influence such segregation: that women are more caring, supportive and nurturing; that women are better communicators; that women are ‘better organized’; and that men are more creative because they are less bound by rules.

## Introduction

This chapter is underpinned by the following assumptions: sexism in society and culture creates conditions of profound difference and inequality between men and women; this has marked effects on all forms of work; and that such difference and inequality are likely to take particular forms in the cultural industries, because of certain distinguishing features of the cultural industries vis-à-vis other industries. We draw on some secondary, statistical sources, but ours is primarily a qualitative approach aimed at understanding the experiences of workers, and their understandings of these experiences, and so we do not focus on statistically demonstrating this inequality in its various forms. Instead, we focus on a particular aspect of how gender inequality makes it harder for women to have good experiences of cultural work than men: division of labour in the cultural industries according to sex.

According to a census produced by the UK government's skills training body, Skillset (2010), about 42 per cent of the UK ‘creative media industries’ workforce is female, compared with 46 per cent in UK industry as a whole. However, this masks a considerable disparity between industries, with very low levels of female representation in the interactive content (5 per cent) and game industries (6 per cent), high levels in industries such as book publishing (61 per cent female – the only subsector where female employment was above 50 per cent) and radio (47 per cent). Two other industries that we discuss below were at or above the national average, and therefore relatively ‘feminized’: television (41 per cent women) and magazine publishing (48 per cent women). A third industry that we discuss below, the music industry, was not included in the Skillset census. But a figure circulated by the UK rights society, PRS for Music (2013), and attributed to research conducted by another Skills Council, Creative and Cultural Skills (2012), cites a figure of 32 per cent women and 68 per cent men in the music industry, including the recording and live sectors. These figures almost certainly represent increases on previous eras.

Behind these employment statistics regarding the concentration of women and men lurks a different but related problem: what is generally known by researchers as occupational and job *segregation* by sex – which we will call sexual work segregation for short. There is a tendency in perhaps all existing societies for some occupations and jobs to be strongly associated with women and some with men, though there is significant cultural variation in the categories. Examples of occupations associated with women in Europe and North America in recent decades include nursing, primary teaching, hairdressing and other ‘beauty work’, and certain kinds of manufacturing work involving ‘manual dexterity’ (Bradley, 1989). Occupations strongly associated with men include mining, driving, professional catering, plumbing and car sales. With the entry of more women into the workforce over the last forty years in many countries, some occupations and jobs have become ‘feminized’ – Wharton (2012: 194) names public relations, systems analysis, bartending, advertising and insurance adjusting as examples. But ‘feminization’ rarely refers to a predominantly male occupation becoming predominantly female. Instead it tends to denote an increase in the concentration of women within that occupation. Segregation, as Browne (2006: 5–6) emphasizes, is not the same as inequality. It can be thought of as having vertical (inequality) and horizontal (difference) components. As Browne points out, however, ‘segregation tends to possess a messy combination of both horizontal and vertical dimensions’ (2006: 5).

There is a considerable research literature on work segregation by sex (eg Bradley, 1989; Blackburn *et al.*, 2001; Hakim, 1979). Most books on gender and work devote some space to it. ‘Segregation’ is not necessarily used to mean full segregation – it is a relative concept, and takes different degrees in different occupations and jobs (and also in organizations – see Halford *et al*., 1997 for relevant discussion). We use the rather awkward phrasing ‘by sex’ to avoid confusion with the issue of ‘sex work’ (such as lap dancing and selling sexual services). Our concern is not sexuality, though of course this has an important role to play in sex inequality in the workplace. Rather it is the sexed division between men and women, which of course is hugely affected by gender. Like Browne (2006: 3), we prefer the term ‘sex’ to that of ‘gender’ in the context of goals of equality and justice, because we seek equality between men and women rather than equality along the dimension of identification with socially constructed notions of femininity or masculinity. As Browne points out, ‘this would be neither possible nor particularly desirable in the pursuit of any practical notion of societal justice’ (2006: 3). This is in no way to suggest that gender is unimportant; this is emphatically not based on a desire to return to biological or Lacanian theories of sexual difference. Gender is fundamental to our analysis below, as it is to Browne's. But equality of men and women, regardless of their biological sex, rather than the hazy and confused concept of gender equality, is the goal. (Equality of transgendered people with other people is a separate issue, but is absolutely compatible with that goal of sex equality in our view.)

The reasons why feminists (of both sexes) should be concerned with work segregation by sex are, surprisingly, rarely made explicit. We will suggest some here. First, it is strongly linked to inequality. For example, jobs and occupations carried out by women rather than men tend to be paid less. This is made strikingly clear when pay rates between countries where a certain occupation is dominated by men (such as dentists in the United States) are compared with a country where women have a more equal or even dominant share of jobs in that occupation (such as dentists in parts of Europe). Pay tends to be considerably lower for the same job in the latter case. Second, work segregation by sex limits the autonomy, freedom and recognition accorded to individual women and men. When a woman has a set of talents that would make her well suited to thrive in a particular occupation, but that occupation is considered ‘male’, then this makes it much more likely that she will not pursue that occupation. The same is true of men who wish to pursue occupations that are gendered female, but given the extra limitations on women entering labour markets, occupational segregation as a whole disadvantages women more than men, and this exacerbates inequality. Third, work segregation by sex limits collective flourishing, because it leads to a situation where it is harder for people to match their talents to occupations, thus inhibiting the way in which people's talents might serve the common good. Fourth, work segregation by sex both draws upon, and in turn contributes to, social ‘stereotypes’ which limit women and men's freedom and recognition – reinforcing the problem of gendered occupational segregation. We return to this important issue of stereotypes in what follows, as it has a considerable bearing on sex segregation in the cultural industries which is itself the key source of social representation, whether stereotyped or otherwise.

There has been a great deal written on work segregation by sex, but very little of it concerns the cultural industries. One major exception is Browne's (2006) fine study of ‘vertical occupational sex segregation’ at the BBC. But Browne, who is not a cultural analyst, pays no attention to how the specific nature of the BBC as a culture-producing organization might be the source of factors that influence sex segregation dynamics there – a major focus of our contribution here. In turn, very little of the considerable literature on cultural production has addressed sexual work segregation in any detail. Some of the rare exceptions are discussed below (such as Banks, 2009; Frölich, 2004; Nixon, 2003). Although there are many other important aspects of sexual inequality and gender dynamics in cultural work, our theme in this chapter, then, is work segregation by sex, which of course is one aspect of the more general problem of division of labour by sex. In the next section, we provide a brief overview of our research methods. We then outline some of the main forms which, according to our observations, work segregation by sex takes in the cultural industries: the high presence of women in marketing and public relations roles in the cultural industries; the high numbers of women in production co-ordination and similar roles; the domination of men of more prestigious creative roles; and the domination by men of technical jobs. Next, we move from problems to possible explanations of them: what gender dynamics drive such patterns of work segregation by sex? Here we consider some of our interviewees’ explanations of such segregation in their cultural workplaces, reflecting on what this tells us about the effects of stereotypes, or prevailing discourses, concerning the distinctive attributes of women and men. A theme that emerges from the discussion, which we briefly consider at the end, is as follows: to what extent does the attribution of particular strengths and styles (such as an ability to deal with emotion and intimacy) actually serve to limit women's quality of working life?

## Methods: interviews and participant observation

This chapter extends the analysis of the quality of working life in the cultural industries presented in our book *Creative Labour* (Hesmondhalgh and Baker, 2011) by drawing out the gendered dimensions of this work. Theoretically, the book sought to bring together the ‘turn to cultural work’ in recent social and cultural research (Banks, 2007; Ross, 2009) with contributions to the sociology, anthropology and philosophy of work, and thereby address the question ‘to what extent do the contemporary cultural industries offer good work?’ The simplicity of that adjective ‘good’ represented a deliberate attempt to evoke the importance of ethics and normativity. The turn to cultural work, we were suggesting, would benefit from greater clarity about evaluation of working life in the cultural industries, and therefore about what reforms might be argued for. Empirically, the book drew on interview and participant observation research conducted in three industries – music, magazine publishing and television – in order to provide a spread of case studies. We also drew extensively on other sources to contextualize those industries, and to understand their specific organizational dynamics.

Gender was a significant concern from the start. In our interviews and case studies, we attempted to balance the proportion of men and women, and to talk to workers at different levels of the industries we studied. We paid careful attention to gender in coding the results, and intended to write a separate chapter on gender. While gender issues appeared at various points throughout the book, such as our chapter on emotional and affective labour in the cultural industries, we did not find time to integrate our findings with existing theoretical and empirical research on gender and work in general (or with the very small number of studies on gender and cultural work). This chapter therefore seeks to remedy this fault at least partially, by drawing on our empirical material, and on previous research on gender and work, gender and cultural production, and cultural production and work.

The fieldwork for the study was conducted in 2006–7 (and was funded by the Arts and Humanities Research Council). Both of us have continued to research the cultural and media industries and, in our view, while these industries have continued to change, as they always do, they have not changed so much that our fieldwork does not cast interesting light on present realities. The fieldwork was done entirely within England. We make no claims about the international generalizability of the data. However, based on our familiarity with cultural industries in other Anglophone countries, we believe it likely that some of these patterns would be reproduced elsewhere in the (over) developed world.

## Forms of segregation by sex in the cultural industries

In the cultural industries, as in many other sectors, the tasks most often carried out by women rather than men include public relations and marketing. In 1984, Steward and Garratt noted that ‘In the big, happy record company family, a woman's place is in the press department’ (quoted in Negus, 1992: 115). Things have changed somewhat – there are other roles that women have begun to take on. But across all three of the industries that we studied (television, magazine journalism and music) many of the marketing and PR staff we talked to were women, working in departments where women were in a majority. As Negus (1992) explains in relation to the UK recording industry, it was not always this way: in the early 1970s, nearly all ‘publicists’ were men. PR and marketing were among those occupations that were feminized in the 1970s, both inside the cultural industries and more generally. PR and marketing can be seen as cultural occupations that exist in many – indeed most – industries and in many firms, including in the cultural industries themselves. Aldoory (2005) claimed in 2005 that the PR profession in the USA had developed to a point where over 70 per cent of practitioners were women, though as Frölich (2004) points out, an even higher proportion of trainees in PR and journalism are women, and there is evidence that women leave these industries much more than men.^1^ The feminization of journalism (Franks, 2013) has almost certainly further contributed to the feminization of PR, as many journalists migrate to work in the often more secure and better-paid world of public relations.

A second area of cultural work that is markedly female in the composition of its workforce is, broadly, those types of work concerned with the co-ordination and facilitation of production. And this relates closely to a third area of occupational segregation: that ‘creative’ jobs tend to be taken by men. On visiting an independent television production company, one of us noticed that the first half of the office area, nearest to the reception, was all male. Our interviewee said ‘this is the creative side’ and told us that the other half of the office area, which was entirely female, was for ‘production’ (Esther, Interview 40). This was by no means atypical in television production, and importantly, the creative side is more prestigious. One company that we researched in some detail was based around two men ‘in the business of actually putting the programmes together’ and ‘everyone else who facilitates that process is female’ (Gary, Interview 24). A female documentary producer and production manager told us: ‘There are far more male directors than women and there are more women enablers, kind of bossy boots. Totally, totally crap that is, isn't it?’ (Lilith, Interview 43). Such hierarchization is also apparent in the case of public relations and marketing, which, like production co-ordination, are less prestigious occupations within the cultural industries than are creative roles.

Nevertheless, some interviewees noted shifts in segregation by sex. ‘Creative management’ roles in television seem to be increasingly occupied by women – especially the key roles of commissioning editor or commissioner (though this is partly dependent on genre, as indeed are many of the phenomena that we observed). These are rather more managerial than they are creative – the core of the job is to organize and handle the creative outputs of others. The job is not dissimilar to that of the commissioning editor in publishing, a role that was feminized relatively early, in the 1970s and 1980s (see Henry, 2009).

The gendering of creative and ‘non-creative’ roles echoes findings in research on other cultural industries that we did not have the opportunity to study, such as advertising.^2^ Sean Nixon (2003) cited figures showing that, by the year 2000, there was a considerable range in the presence of women in the various roles in advertising: 60 per cent of finance and administrative workers were women, 54 per cent of account handlers (up from 33 per cent at the start of the 1990s) and 44 per cent of media planners/buyers. But only 18 per cent of creatives were women, and this percentage actually declined in the 1990s. Combined with problems for women in gaining promotion, endemic in most industries (and which we will discuss below) this in turn meant that very few women achieved the position of creative director. Yet, because marketing had become increasingly feminized, as discussed above, the marketing managers to whom advertisers were presenting were often female: an imbalance of which agencies were strongly aware. According to figures cited by Nixon (2003: 96), 50 per cent of marketing managers were female by the end of the 1990s.

There is a fourth form of work segregation by sex in the cultural industries, which will perhaps come as no surprise, because of the long and problematic relationship between gender and technology (see Wajcman, 2011): as in other industries, men tend to dominate technical and ‘craft’ jobs, such as camera operators and editing in television, engineering and ‘road managers’ or roadies (technical staff handling equipment) in the music business. What is more, as Miranda Banks (2009) points out, craft and technical occupations associated with women, such as costume design, tend to be relatively unrecognized and undervalued. This can happen to the degree that such occupations are not even recognized as involving craft or technical skills at all.

In pointing to the marginalization of women from key creative roles, we should be wary of simplification about the relations between ‘above the line’ creative and ‘below the line’ technical and craft occupations. While creative roles might sometimes be more prestigious, and more recognized publicly, actual creative workers receive very unequal rewards and have very different levels of power and autonomy from each other. Creatives are highly hierarchized, in ‘winner take all’ markets where the successful few are disproportionately rewarded (see Hesmondhalgh, 2012, for discussion of this phenomenon). Technical and craft jobs can in fact be of higher quality, and receive greater levels of union protection than ‘creative’ ones and can be relatively prestigious, especially compared with facilitation and marketing roles. These issues are important in the present context because technical and craft jobs tend to be taken by men – and there may be divisions within the creative jobs, whereby occupations with high numbers of women, such as acting, are prone to uncertain work conditions. We are likely to understand the complexities of segregation by sex better, the more we drill down to specific job levels, rather than looking at occupations or occupational groupings (such as creative or craft workers) as a whole.

## Explaining work segregation by sex in the cultural industries: caring and communicating

So, we have presented a number of ways in which work segregation by sex is manifested in the cultural industries. How, though, do we *explain* such patterns? To ask such a question invokes the broader problem of explaining work segregation by sex in general. Anker has discussed how some dominant social science theories, notably neo-classical, human capital and institutional labour market models, tend (a) to treat occupational sex segregation as though it is the same thing as sex-based pay differentials, when it is not; (b) fail to provide an explanation of how occupational sex segregation comes about.^3^ Anker (2001: 139) claims that feminist gender theory ‘makes a valuable contribution to explaining occupational segregation by sex by showing how closely the characteristics of “female” occupations mirror the common stereotypes of women and their supposed abilities’. He provides a list of such ‘stereotypes’ and the occupations that tend to be affected by them. Some of them are positive, such as the idea that women have a caring nature, that they are skilled in domestic work, or that they have greater manual dexterity, trustworthiness and attractiveness. Such views feed the gendering of occupations such as nursing, teaching, social work, hairdressing, dressmaking, book-keeping, reception and shop assistant work, and so on. Some are negative, such as ideas that women are less able to supervise others, that they have less physical strength (many women have greater physical strength than many men), that they are less able in science and maths, that they are less willing to travel, or to face danger and use physical force. This affects the gendering of occupations such as management, mining and construction work, engineering and transport, and security work. Then there are other, more ‘neutral’ or ambivalent characterizations of women as being less inclined to complain, more willing to take on monotonous or repetitive work, and more interested in working at home. These tend to push women in the direction of jobs that are low paid, unprotected and often repetitive.

The term used by Anker, ‘stereotype’, merits some consideration. Questions of culture, meaning and discourse have been an important element of feminist theory in recent decades (see Fraser, 2013, for an incisive discussion of this issue). The concept of stereotyping may seem to some rather basic compared with sophisticated debates about issues such as the gendering of language itself. Certainly, it has fallen from favour in media and cultural studies over the last 30 years (though see Pickering, 2001, for a defence and clarification of the concept) and in feminist media studies. We would argue, along with feminists such as Robeyns (2007), that stereotyping is an important concept for considering the way in which prevailing and repeated categorizations might influence the treatment of individuals and groups, provided it is applied with sufficient critical rigour, and provided it is combined with other factors in any explanation.

Wharton (2012) discusses two other factors identified by researchers as causes of sex segregation: workers’ own preferences, shaped by their own histories; and effects of workplace processes such as recruitment and assignment of roles. Wharton, who does not explicitly discuss stereotypes, argues that there is evidence that the effects of early ‘socialization’ are sometimes exaggerated, and the importance of employers’ actions consequently downplayed.^4^ Policy is also a vital consideration, as Browne (2006) shows. All these factors are important and need to be combined with the effects of stereotypes in understanding sex segregation in the cultural industries. But here, for reasons of space, and because of the nature of our own data, we focus on gender stereotypes, or prevailing discourses about the characteristics of women and men, as potential explanations of sex segregation.

Let us start from the case of PR and marketing. Observing the relatively high numbers of women in recording industry PR, Negus (1992: 114) suggested some of the reasons for this phenomenon: PR work ‘involves the employment of skills which have traditionally been associated with women rather than men: looking after sensitive artists, maintaining personal relationships, providing support, and acting as a facilitator and catalyst’. The idea that women are more capable of caring, supportive and nurturing work than men (already mentioned above in relation to Anker's list, and widely recognized as a factor in understanding women's work) may lie behind the presence of women in PR. But related ideas were also invoked by some of our interviewees as a factor behind other forms of work gendering. Here, for example, is how one woman we interviewed sought to explain why documentary researchers were often women:

I think a lot of women tend to put people more at ease. They're not so threatening in some situations. They can make themselves quite vulnerable, just physically vulnerable. They're smaller. I think each film dictates its own approach. It's a journey and every film makes itself in a way. So maybe a good woman filmmaker would use whatever she needed to use. I think any good filmmaker really, but some of the men I know seem to have more of an agenda on their films and more of a kind of bigger view. (Lilith, Interview 43)

Closely linked to this idea of women as more caring, sympathetic and able to put others ‘at ease’ is the association of women with greater communication and presentational skills, which supposedly allow them to maintain personal relationships and prevent conflict. Here is an explanation by a male executive producer of factual television of why more and more women were working in this genre, where he claimed that talking to people ‘in a relaxed way’ was a requirement:

I think the reason it has become very female is because women are also obviously better listeners. They have been brought up with a stronger emphasis on communication, listening. So maybe it's a gender stereotype forced upon people, but the fact is by the age of 20, 25, they are much more socially competent than men are. So if you are in an area which is predominantly people based and finding out about people and getting people to talk about themselves in a relaxed kind of way, then women tend to be better at that. (Kieran, Interview 20)

This kind of explanation may go some way to help understand the predominance of women in jobs and occupations that involve ‘enabling’ or ‘co-ordinating’, as discussed above. Whether women really are better communicators or listeners is a moot point. The key issue is that people working in television and other cultural industries have come to see gender in this way, and this has opened up a space for women, and perhaps closed one down for men.

We detected another ‘stereotype’ or prevailing discourse in operation in the cultural industries, which seems to have been discussed relatively little in social scientific studies of work in general, at least as far as we have been able to discern. This is the idea that women are better organized, and that they take greater care over procedure and so on. So roles such as production manager, production co-ordinator and production assistant were conceived by some interviewees, including relative newcomers working in creative fields, as ‘female roles’ (Gary, Interview 24). One of our interviewees used this idea to discuss why, as mentioned earlier in this piece, the role of programme commissioning in television was increasingly taken by women:

So you have gender models. The two different genders overlap a lot but they also have different ways of succeeding. Women offer by and large a variation of skills. Men are more mercurial, often more difficult to handle. Women are often very steady, solid and organised. You can still have very creative women and very uncreative women, and very creative men and very uncreative men, but they are different. I mean these are gross generalisations. (Malcolm, Interview 37)

One head of production attributed the dominance of women in production co-ordination to women's ‘ability to multi-task’ and be ‘very good organisers’ (Esther, Interview 40).

Such organizational skills were explicitly contrasted by some of our interviewees to the kind of attributes that were supposedly necessary to be good in ‘creative roles’. So one interviewee suggested that careful co-ordination and facilitation were not attributes of a ‘good director’ (which is a ‘creative’ rather than an ‘organizational’ role in factual production, where he worked). His reasoning was as follows:

I suspect women are better organisers and want to feel that something is under control and well managed. Your good director, the one that's different, is actually the one who is going to want to put a wheel off the wagon and see what happens and take a risk. … That is something you notice more with reckless males than you do with incredibly well organised and nice women. (Kieran, Interview 20)

It would only be fair to point out that Kieran was trying to explain the common sense of the industry, and how it contributes to work segregation by sex (though this was not the term he used). The line between observing common stereotypes and tendencies and seeming to affirm them is often very thin.

This can be seen when comparing such discourse with women's explanations of what they feel they, as women, can valuably bring to a workplace. One female managing director explained why she thought that ‘women are incredibly good in television’ in ways that relate to the above notions that women have skills which nurture other people's talents:

We have loads of advantages. We are collaborative and we love working in teams. I mean, women really actually love working with other people, and they are very good at getting things out of other people and making them work to their best abilities. Women actually enjoy that I think. I'm not saying that men don't, but I think women particularly do. (Ingrid, Interview 11)

Is this reaffirming the stereotype that leads to work segregation by sex, or is it celebrating women's distinctive virtues in a way that opens up new spaces for women's employment? Or might it even be both?

In a rare and thoughtful discussion of such problems in relation to cultural work, here in the context of the gendering of journalism,^5^ Frölich (2004: 71) conceded that ‘worse things could happen to women’ working in these fields ‘than to be casually regarded as being able to communicate better simply because of their friendly, polite, consensus-oriented behavior’. However, she also suggested that such social sensitivity may derive from an effort to deal with a lower social status – these are ‘tools that would enable them to survive and function in society’. This may have some validity, but not all subordinates are polite and sensitive, and not everyone in ‘higher’ social groups shows the opposite traits, partly because, in spite of what Frölich (2004) implies, not all social behaviour is competitively aimed at achieving personal goals. Nevertheless, Frölich may be right to point out how the possession by many women of ‘communication skills especially oriented toward consensus and dialogue’ (2004: 72) allows some women access to communication professions at the entry level, but does not necessarily influence how long they stay or how far they advance. ‘Perhaps’, she suggests, ‘the very attributes that get women into the communications sector – sensitivity, caring, honesty, fairness or morality – are also associated with a lack of assertiveness, poor conflict management and weak leadership skills’ (2004: 72). Yet it would surely be unfortunate if feminists responded to such a trap by arguing against the presence of these attributes among women. Frölich's (2004) term, ‘the friendliness trap’, seems an apt one here.

What of the clustering of men in the more prestigious creative roles, across many different cultural industries, noted earlier? What might explain this? Nixon's (2003) important study of advertising argued that the gendering of creative roles was protected and reinforced by a legacy of associations between masculinity and creativity. He drew on the work of art historian Griselda Pollock and others (see Parker and Pollock, 1981), who showed how features attributed to the creative artist – ‘dependent, insecure, expressive, over-emotional and prone to infantile egotism’ – placed the male artist at odds with more conventional versions of masculinity, but gained their power from ‘being set simultaneously against representations of femininity that suggested that women could at best express taste’ rather than ‘true’ creativity (Nixon, 2003: 100). This notion of masculinist creativity was apparent in the culture of creative departments, but it co-existed with a somewhat different masculine ethos of ‘the creative as aesthete and man of taste’. But this mix of masculinities produced working cultures in which childishness and laddishness were valued (and Nixon was doing his research at a time when the figure of the ‘new lad’ was hegemonic in UK culture, partly as a result of developments in magazine publishing, and the rise of a new generation of men's magazines), and women were often seen as responsible for mothering and nurturing. This of course served to marginalize women from creative roles, and the ‘mothering’ roles became associated with account planning and other co-ordination roles. More recently, Proctor-Thomson (2013: 147) has discussed how the seemingly high value placed on gender diversity in the digital media sector in fact serves to ‘exclude particular forms of difference and diversity from those considered to hold creative potential’.

Needless to say, perhaps, the kinds of segregation by sex that we have been describing here resulted in situations that were not welcomed by women. ‘Sometimes’, one head of television production put it, ‘I feel I'm like a mother with hundreds of children, and that can be quite frustrating’ (Esther, Interview 40). Yet for other female interviewees, caring and nurturing were valued as their distinctive contribution to cultural work. One successful female artist manager described how what she saw as a distinctive ‘female management style’ allowed her to mark out her own place:

I would say that female management style is very much artist led … I really actually think my interpretation of management is to become a translator for that artist. So you are basically taking their vibe, their whole ethos, their philosophy, and you are trying to preserve as much as possible and translate it into a package that makes sense to the consumer. (Hannah, Interview 42)

This was in contrast to a more mechanical, less emotional male style that ‘set[s] all your affairs by conveyor belt’ (Hannah, Interview 42). It also contrasts with an almost legendary history of macho behaviour on the part of artist managers, supposedly in the service of their clients (see Summers, 2013). In valuing caring and nurturing styles as elements of their own and other women's work, are these women unconsciously reproducing stereotypes that then constrain women in the cultural workplace? That would surely be a harsh judgement. Education and employment policy needs to open up cultural workplaces to make all kinds of work available to women as well as men. And reasonable, constructive (‘caring’) approaches need to be more than just a niche that women feel they can occupy. Men should feel obliged to aspire to such behaviour too.

Some of the context for understanding gendered divisions of labour in the music industries, and what women working there have had to face, was provided by one music journalist:

The music industry is still an incredibly sexist industry. I don't care what anybody says, I really would on the record say it's a blokes’ industry and girls are press officers or stylists or groupies and it still is ‘my best mate is my manager’. Somebody at quite a big independent label decided she was going to become a manager and they just went ‘what do you want to become a manager for? Do you just want to shag loads of bands?’ and she went ‘no, I want to be a manager’. But still even within the independent community, there is that belief that a girl working in the music industry is just a glorified groupie. I think slowly that is changing. There are obviously a few high profile females, certainly in the publishing world.^6^ I think the publishing world is perhaps not that bad as the record industry and there are a few powerful female managers and stuff like that. (Niall, Interview 17)

This suggests the same association of creativity with masculinity as discussed by Nixon (2003) in relation to advertising. Here, though, the dynamics are primarily sexual rather than infantile/maternal. Women's roles are portrayed as sexually subordinate. And when Niall points here to the greater presence of women in ‘the publishing world’ – by which he means ‘music publishing’ – he draws attention to the way in which work segregation by sex can be manifested at the level of entire industries, not just occupations, jobs and organizations.

## The baby and the bathwater

Associations of various modes of masculinity with creativity, then, serve to marginalize women from the more prestigious creative roles and even sectors in the cultural industries. This, as we have shown, is just one way in which work segregation by sex occurs in the cultural industries. Others include the assignment to women of work involving the need for consensual and caring communication, and co-ordination. As we suggested above, however, it would be a mistake to argue too strongly against the high evaluation of such skills by women. Rather, we need to argue for a greater respect for such qualities, in both women and men. Similarly, when it comes to the gendering of creative roles, it would be a mistake to respond to the gendering of ‘creative roles’ by seeing all positive evaluations of ‘creativity’ as encumbered by sexism and patriarchy. For the view that creativity should be protected from commerce, that commerce should have boundaries, is an important way in which to protect the relative autonomy of aesthetic experience and public knowledge. They can and should be untied from dubious gender politics. We need to examine *how* the commerce-creativity division of labour becomes attached to gendered divisions of labour, and recognize a much more varied set of modes of creativity, moving beyond dubious connections of creativity with infantilism and sexuality. As Edwards and Wajcman (2005) suggest with respect to stereotypes regarding leadership and management, dichotomies of hard and soft need to be broken down. Gender stereotypes matter hugely in the division of labour by sex.

## Notes

1 See also Beetles and Harris's (2005) report on studies of the feminization of marketing.

2 It also echoes Banks and Milestone's (2011) careful consideration of how traditional gender roles both persisted and were being questioned in the digital ‘new media’ sector. Their references to theories of individualization and reflexivity help make links with ‘service sector’ jobs more generally, an issue we do not have space to consider here.

3 Jude Browne's (2006) more comprehensive review criticizes human capital and neo-classical models and also a range of other theories, including ‘preference’ models (such as those of the feminist sociologist Catherine Hakim, 1979), patriarchy models (such as those of Sylvia Walby, 1990), Carol Gilligan's (1982) ‘different voices’ concept, and biologically based theories of dominance (such as Steven Goldberg's, 1993).

4 These broader factors would be best approached by ‘life history’ studies (see Taylor and Littleton, 2012), rather than participation observation and interviews – the methods we used.

5 Recent discussions of cultural work have not paid sufficient attention to the formidable literature on working conditions within journalism. Although we made some efforts in this direction in our 2011 book, we did not go nearly far enough.

6 Music publishing does not, as the name might suggest, refer to the publishing of sheet music (a marginal aspects of the music business for decades now) but to a sector of the music business involving the administration of the ‘rights’ that inhere in composition and the secondary rights that derive from ownership of the rights to songs. It is generally seen as more administrative, more businesslike, less creative than the more glamorous recording industry. The latter has been much less ‘feminized’ than the former.
